# Editorial Notes

**Published:** 1902-03

**Authors:** 


### Editorial Notes.

**A University of Bristol.**—We believe that it is not unreasonable to hope that the movement in favour of establishing a University for the South and West of England was distinctly advanced at the Annual Dinner of University College Colston Society, which was held in the large hall of that institution on the 5th of February. The great philanthropist, Edward Colston, in memory of whom the Society has been named, was a pioneer in the education of the people; it is therefore fitting that the dominant thought expressed in the speeches at this annual gathering should have been the urgent need of a University for the part of the United Kingdom of which Bristol is the centre.

The toast of "The King" having been loyally honoured, Dr. Shingleton Smith submitted that of the "Ministers of all Denominations"; and while reminding those present that the old and flourishing Medical School of Bristol was the germ from which University College was raised, gratefully acknowledged the active and prominent part played by the clergy of various denominations in founding that College, mentioning especially Dean Elliott, the Bishop of Hereford, the Rev. Dr. Jowett, and the Rev. Dr. Gotch.

The Lord Bishop of Hereford, in welcoming the guest of the evening, Mr. Haldane, K.C., M.P., pointed out that he was a member of a distinguished University of Scotland, and a Scotch University was pre-eminently a University for the people; and if he understood the aim aright, it was that the Bristol University should be a University for the people. But Mr. Haldane was likewise a member of a German University. All knew that whilst in England they had been slow and conservative in the establishment of Universities, in Germany they had multiplied, and this perhaps, more than any other fact, accounted for Germany forging ahead.

Mr. Haldane, K.C., M.P., stated that "the extension of the University movement in this country touched the very heart and the life of this country. The Educational System of the country would never be put on a proper footing until the whole system of education—secondary, primary, and technical—in each district was permeated from the top. That meant we must have more Universities. The great cities of Manchester, and Liverpool, and of Yorkshire were seeking to have their own Universities, that their manufacturing and social life might be permeated by that influence which a great University only could furnish. So it had been at Birmingham, and in the determination of Liverpool to have a University of her own they had only a sign of the advance of the times. Now the West of England! They were without anything of the kind. It seemed to him there was probably no part of the United Kingdom which was really more in need of the concentration of academic purpose which a University alone could give than the West of England. They had that admirable institution, the University College in Bristol; they had that great and most efficiently organised institution, the Merchant Venturers' Technical College; but they lacked what nothing could take the place of—the influence of the University itself.

"But Bristol must try to bring within its ambit as much as possible of the rest of the country of which they were the centre. There was the University College at Reading, with a very distinguished principal; they had Southampton, Exeter, and the possibilities of development in the future in such great towns as Plymouth and Devonport. They had to consider how they could best find a pattern of University institution which would succeed in embracing the elements which they desired to bring in, and leave intact and outside the elements which belonged properly to secondary education. It seemed to him the time was ripe for a movement such as this in the West of England."

We are pleased to record that Sir William Hy. Wills, Bart., has offered £1,000 to the funds of University College, provided that four other contributions of a similar amount are promised within a limited period, and that Sir Fred. Wills, Bart., M.P., has guaranteed one of these thousand pounds.

The authoritative expressions of opinion which we quote in brief abstracts, were warmly endorsed by the Lord Mayor and by Mr. Howell Davies from the standpoint of commerce; and though our readers all belong to one section of the public, they must feel that the standing of the medical profession in the West of England will be raised by having a University within their district, and that such a necessary and happy consummation can only occur by the linking together of the various interests of professional and commercial life.

The striking changes which are impending as regards University extension in the Northern counties shows that there exists in the North a thorough awakening of local interest in the advancement of higher education. If Liverpool is to have a University of its own, if mere partnership in the Victoria University does not suffice for its needs, then the latter will naturally become the University for Manchester, and Leeds will aim to be the centre for the whole county of York. It appears that the possession of a University of its own is a thing which strikes the civic fancy, and so Birmingham and Liverpool have resolved that they will be absolutely independent. The Corporation of Liverpool are proposing that a considerable annual grant to the proposed civic University shall be provided out of municipal funds; in Birmingham this does not appear to be needful, as the University, of which Mr. Chamberlain is the first Chancellor, has had no lack of financial support. In Bristol, we can scarcely expect that the example of Birmingham, or even of Liverpool, will be quickly followed, but it is time that the question of University extension in the West of England should be taken up with spirit; and if Bristol does not wish to act by itself as the Northern cities are doing, Mr. Haldane has shown how a Western University may be provided by a combination of all the forces of the district.

Bristol is proposing to expend something like £2,000,000 sterling in Docks extension, and she has already enormous commercial interests. But we venture to assert that it is a short-sighted policy which launches on great commercial enterprises while neglecting the obvious necessity of providing facilities for that wider education which is essential now, and must be more so in the immediate future, which alone will enable the community at large to turn their commercial opportunities to the greatest advantage and so reap the benefit of their spirited policy. If this applies to Bristol, it does so in no less degree to Southampton, and to Plymouth, and other great centres in the South and West of England, though it is not cities and centres alone which must be taken into consideration, but also the great agricultural industries which form the staple employment of millions of the inhabitants. Let us therefore hope that the South and West of England will not long remain the only part of the United Kingdom without a University suited to its needs.

**Smallpox in Bristol.**—As yet, the epidemic of smallpox in London has been so successfully managed that the disease has taken little hold of other centres. As regards Bristol, we have ascertained that the facts are quite simple, and are as follows:—

(*a*) A man of 29. L. J. K., A.B. on steamer trading from London, arrived at Avonmouth, 19th December, 1901, on the fourth day of eruption of a very modified attack of smallpox. He was isolated the same evening, and discharged cured from the Hospital Ship on Jan. 16th, 1902. No cases are known to have resulted from this one, which was promptly diagnosed and reported by Dr. Hedley Hill.

(*b*) A man of 28. P. H., A.B. on a steamer from Portland, Maine, U.S.A., arrived at Avonmouth 6th January, three days ill with primary hemorrhagic smallpox (malignant or black smallpox), was admitted to the City Hospital on same evening, and died on 7th January. No cases are known to have resulted from this one, which was reported from the General Hospital.

For these facts we are indebted to the Medical Officer of Health, Dr. D. S. Davies, who makes the following comment thereon:—"It is curious that both cases escaped recognition by the master of the vessel, the first being so mild that the sailor continued to perform his ordinary duties, the second so unusual that the idea of smallpox was not presented to the lay mind. Notification was thus omitted, and the patients made their way by train into the City. Fortunately they fell into good hands, so that isolation of patients, disinfection of train, cab, and re-vaccination of contacts was secured very early, and apparently with complete success. These introductions by ship frequently happen, and we have become accustomed to expect no result; early and correct diagnosis is the important factor in suppression. Nothing further has occurred."

The subject of **Contract Practice**, its advantages and evils, is ever present, on which an outcry is from time to time renewed, the burden of which is "Fair play for the doctor." The question has been recently brought before us at a meeting of the Bath and Bristol branch of the British Medical Association by Dr. A. Baillie McKee, who thought it would be generally admitted that contract practice is not the best thing for the patient, the medical man, and the profession as a whole. He pointed out that the evils attending it arise from the assumption by the medical man of a *rôle* which does not properly belong to him, through his acceptance of risks which ought to be borne by the societies and clubs. He proposed that the subscriptions of members should be placed in a "Medical Attendance Fund," and the members should be free to choose their own medical attendant, fees being paid for each item of work done. He expressed his belief that this method would be very acceptable to the working man, and if once fairly started would gradually take the place of the present system of contract. Dr. McKee's proposals are expounded at some length in *The British Medical*

*Journal* (Feb. 8th, 1902), and we think that they are worthy of careful consideration and trial.

**Sanatorium Treatment of Consumption.**—As so many West of England medical practitioners have a direct interest in the progress of the Winsley Sanatorium for consumptives, we are glad to report the advances that have been made since our last issue towards the realisation of the scheme. The levelling of the site has been completed, and a large quantity of good building stone has been prepared and stacked ready for use as soon as the Committee have sufficient money in hand to justify their commencing to build. This, however, will necessitate the collection of several thousand pounds yet.

It would seem that not only will the Sanatorium be a model in its construction, but it will furnish an excellent example in its financial aspects; for the Corporation of Bath has undertaken to contribute £500 towards the cost of construction as soon as the building is erected, and to maintain two beds by an annual subscription of £130; and the Great Western Railway Club, Railway employés, promise £400 towards construction, and £130 annually to maintain another two beds.

A deputation from the Executive Committee of the Gloucester, Somerset, and Wilts branch of the National Association, consisting of Dr. Lionel Weatherly, *Chairman*, Sir John Goldney, Dr. Shingleton Smith, Dr. A. J. Harrison, Mr. Nelson Dobson, Dr. Watson Williams, *Treasurer*, Dr. J. Michell Clarke, *Hon. General Secretary*, and Dr. Kenneth Wills, *Hon. Sec.* for Bristol, waited on the Health Committee of the Bristol Town Council, at a meeting held on February 4th, at the Bristol Council House, to urge the desirability of the Bristol Corporation contributing to the cost of construction and the maintenance of three or four beds for cases nominated by the Corporation of Bristol. Of course, as is only right and fitting, these public bodies who contribute thus to the cost and maintenance will be represented on the Board of Governors; but it is well that it should be widely known that counsel's opinion has been taken, and leaves no room for doubt as to the legality of such contributions by municipal corporations. However confident the medical profession may be that the Sanatorium treatment of consumption is beyond dispute successful in its results, it is quite conceivable that public bodies might hesitate to launch out in the large expenditure that a number of separate Sanatoria would entail. The opportunity thus afforded for Bristol and other municipal bodies to have a few beds of their own will provide for their poorer consumptive patients at a relatively small cost, and we venture to assert that experience will demonstrate the economy of the method which saves working men and women from drifting on to the rates. Hence we sincerely trust that Bristol will follow the example of Bath in this matter.

---

